# PD-1 deficiency promotes TFH cells expansion in ITV-immunized mice by upregulating cytokines secretion

**DOI:** 10.1186/s13071-018-2984-4

**Published:** 2018-07-06

**Authors:** Taiping Liu, Xiangyun Cheng, Yan Ding, Feng Zhu, Yong Fu, Xiaohong Peng, Wenyue Xu

**Affiliations:** 1 Department of Pathogenic Biology, Army Medical University (Third Military Medical University), Chongqing, People’s Republic of China; 2 Department of Parasitology, Guilin Medical University, Guilin, Guangxi People’s Republic of China

**Keywords:** Malaria, Infection treatment vaccine, PD-1, Cytokines, TFH cells

## Abstract

**Background:**

T follicular helper (TFH) cells are fundamental for the development of humoral immunity. In our previous study, we found that PD-1 deficiency substantially promoted the expansion of *Plasmodium*-specific TFH cells and enhanced the humoral immunity of ITV (infection treatment vaccine)-immunized mice. However, the underlying mechanism by which PD-1 signaling modulates TFH cells activation remains unclear.

**Methods:**

Mice were immunized with the ITV following the standard procedures. The activation phenotype of CD11c^+^CXCR5^+^ dendritic cells (DCs), the frequency and number of splenic follicular regulatory T cells (TFR cells), *Plasmodium*-specific TFH cells and germinal center (GC) B cells were analyzed by FACS. The levels of serum cytokines were quantified using the cytometric bead array (CBA) and *in vivo* cytokine neutralization was carried out according to a previously described protocol and verified by serum cytokine detection.

**Results:**

We found that PD-1^-/-^ naïve and immunized mice had more TFR cells in the spleen than WT and WT immunized mice. Additionally, CXCR5^+^ DC, which prime TFH cells, were activated at similar levels in ITV-immunized WT and PD-1^-/-^ mice. However, the serum levels of IL-10, IFN-γ and MCP-1 were significantly increased in ITV-immunized PD-1^-/-^ mice, and treatment with an anti-IL-10, anti-IFN-γ or anti-MCP-1 neutralizing antibody *in vivo* markedly impaired the development of TFH cells and GC B cells.

**Conclusions:**

Our findings demonstrate that the modulation of TFH cells by PD-1 signaling is dependent on the cytokines IL-10, IFN-γ and MCP-1 in ITV-immunized mice. These results could facilitate the design of an effective malaria vaccine with the aim of inducing humoral immune responses.

**Electronic supplementary material:**

The online version of this article (10.1186/s13071-018-2984-4) contains supplementary material, which is available to authorized users.

## Background

T follicular helper (TFH) cells, a particular subpopulation of CD4^+^ T cells, are the key cell type required for the formation of germinal centers (GCs) and the generation of long-lasting antibody responses [1, 2]. These cells are characterized by high expression of the chemokine receptor CXCR5, programmed death 1 (PD-1), a master regulator transcription factor (Bcl6), IL-21, and the inducible T-cell co-stimulator (ICOS) [3]. Inducing TFH cell activation is considered to be a promising strategy for improving host immune protection induced by vaccines [4, 5].

Malaria is a major global health burden, causing 212 million clinical episodes and 429,000 deaths in 2015, mostly in Africa in children under five years old [6]. The development of an effective and durable vaccine is considered to be the most cost-effective strategy for the prevention, control and eventual eradication of this disease [7]. The main approach in blood-stage malaria vaccination is to induce antibodies that can prevent merozoites from invading erythrocytes [8]. However, the need for high titers of *Plasmodium*-specific antibodies remains a significant clinical hurdle [9]. Therefore, understanding how *Plasmodium*-specific B cells are activated during vaccination could help guide the design of a more effective malaria vaccine. The infection treatment vaccine (ITV), a whole blood-stage parasite vaccine, induces protective immunity that is primarily mediated by antibodies specific for merozoites surface antigens [10, 11]. By using this kind of whole blood-stage parasite vaccine mode, our previous study showed that PD-1 deficiency substantially promoted the expansion of *Plasmodium*-specific TFH cells, which may enhance the *Plasmodium*-specific B cell response [12]. However, it remains unknown how PD-1 signaling regulates TFH cell activation in ITV-immunized mice.

TFH cell activation is a complex and tightly regulated process. Current studies suggest that TFH cells are primed by CXCR5^+^ dendritic cells (DCs) and fully differentiate into mature GC TFH cells with the aid of cognate B cells [13, 14]. In addition, developing TFH cells could be influenced by environmental cues, including cytokines (such as IL-6 and IL-21) and chemokines (such as CXCL13) [2, 15, 16]. Recently, a subset of Foxp3^+^ Tregs, follicular regulatory T cells (TFR cells) that inhibit TFH cell-mediated humoral immunity, has been identified [17]. Previous studies have shown that PD-1 or PD-L1 deficiency results in the presence of more TFR cells than TFH cells in lymph nodes and potently inhibits antibody production [18]. However, therapeutic *in vivo* blockade of PD-L1 in mice has been found to enhance the differentiation of *Plasmodium*-specific TFH cells and plasmablasts during malaria infection [19]. Therefore, the mechanisms by which PD-1 signaling modulates TFH cell activation remain unclear.

In this study, we found that neither CXCR5^+^ DCs nor TFR cells were involved in regulating *Plasmodium*-specific TFH cells activation in ITV-immunized PD-1^-/-^ mice. However, elevated levels of IL-10, IFN-γ and MCP-1 substantially contribute to the expansion of *Plasmodium*-specific TFH cells in ITV-immunized PD-1^-/-^ mice. Therefore, we have revealed a novel mechanism by which PD-1 signaling regulates TFH cells activation.

## Methods

### Mice and malaria parasite strain

PD-1^-/-^ mice (BALB/c background) were obtained from the Jackson Laboratory (Bar Harbor, ME, USA). Specific pathogen-free BALB/c mice were purchased from the Beijing Animal Institute. All mice ranged in age from six to eight weeks when the experiments were initiated. The lethal strain of *Plasmodium yoelii* 17XL was originally obtained from MR4 (Malaria Research and Reference Reagent Resource Center, Manassas, VA, USA) and maintained as cryopreserved stabilates. All animal studies were reviewed and approved by the Animal Ethics Committee of the Third Military Medical University Institute of Medical Research.

### Immunization

The mice were immunized following a previously described immunization schedule [12]. Briefly, the mice were intravenously (i.v.) injected with 10^6^ *P. yoelii* 17XL-infected red blood cells (RBCs) (*Py*-iRBCs) or a matching number of normal RBCs (nRBCs) (negative control). All mice were then intraperitoneally (i.p.) injected with 100 μl of 8 mg/ml chloroquine (CQ; Sigma-Aldrich, St. Louis, MO, USA) diluted in saline daily for 15 days starting the day of the iRBC injection. The absence of parasites was confirmed by Giemsa-stained blood smears in all treated mice from the beginning of CQ treatment.

### Flow cytometric analysis

Spleens were collected on the appropriate days, and single-cell suspensions of splenocytes were prepared as previously described [20]. To determine the activation phenotype of CXCR5^+^ DCs by evaluating the expression of CD40, CD86 and MHC class II (MHC-II), 10^6^ cells were pre-incubated with anti-CD16/CD32 (BioLegend, San Diego, CA, USA) to block non-specific binding to Fc receptors. Then, the cells were washed and subsequently stained with anti-mouse CXCR5 (biotin; BioLegend), streptavidin (APC; BioLegend), anti-mouse CD11c (percp/Cy5.5; BioLegend), anti-mouse CD40 (PE; BioLegend), anti-mouse CD86 (PE/Cy7; BioLegend) and anti-mouse MHC-II (FITC; BioLegend). To analyze GC B cells, 10^6^ splenocytes were blocked as described above. Then, the cells were washed and stained with anti-mouse B220 (APC; BioLegend), anti-mouse CD95 (PE; eBioscience, San Diego, CA, USA), and anti-mouse T and B cell activation marker (GL-7) (FITC; BioLegend). To analyze TFR cells, 2 × 10^6^ cells were stained with anti-mouse CXCR5 (biotin; BioLegend), streptavidin (APC; BioLegend), anti-mouse CD4 (APC/Cy7; BioLegend), anti-mouse CD19 (percp/Cy5.5; BioLegend), anti-mouse ICOS (PE/Cy7; BioLegend) and anti-mouse Foxp3 (PE; eBioscience) after being permeabilized with a fixation/permeabilization agent (eBioscience). For *Plasmodium*-specific TFH cell analysis, cells were stained for CXCR5, CD4 and ICOS as described above. The cells were also stained with anti-mouse CD19 (Pacific Blue; BioLegend), anti-mouse CD11a (percp/Cy5.5; BioLegend), anti-mouse CD49d (FITC; BioLegend), and anti-mouse Foxp3 (PE; eBioscience) or anti-mouse Bcl6 (PE; eBioscience) and anti-mouse Foxp3 (PE/Cy7; eBioscience) after being permeabilized. The cells were analyzed on a FACSCanto II instrument (BD Biosciences, San Jose, CA, USA), and the data were analyzed with FlowJo software.

### Serum cytokine detection

The levels of the proinflammatory cytokines IL-6, monocyte chemoattractant protein-1 (MCP-1), IFN-γ, TNF-α, and IL-12p70 and the anti-inflammatory cytokine IL-10 were quantified in serum samples using the cytometric bead array (CBA) Mouse Inflammation Cytokine Kit (BD Biosciences), according to the manufacturer’s instructions. Briefly, mouse inflammation standards were prepared with 2 ml of assay diluent and six mouse inflammation capture beads were mixed thoroughly. Then, 50 μl mixed capture beads were incubated with the same volume of mouse inflammation standard dilutions or each samples. Fifty microliters of the mouse inflammation PE detection reagent was added to all assay tubes and the assay tubes were incubated for 2 h at room temperature, protected from light. After being washed with washing buffer, samples were analyzed on a FACSCanto II instrument (BD Biosciences), and the data were analyzed with FCAP Array software.

### *In vivo* cytokine neutralization

To neutralize MCP-1, IFN-γ or IL-10 *in vivo*, mice were respectively i.p. injected with 200 μg (per mouse) of a neutralizing mouse anti-MCP-1 mAb (BioXcell, clone: 2H5), anti-IFN-γ mAb (BioXcell, clone: XMG 1.2), anti-IL-10 mAb (BioXcell, clone: JES5-2A5) or control Ab every other day starting from one day after the last CQ injection until sacrifice, according to a previously described protocol with minor modifications [21]. Cytokine depletion was verified by serum cytokine detection as described above.

### Statistical analysis

The data were analyzed using GraphPad Prism version 6 software. Nonparametric tests (Mann-Whitney test) and two-way ANOVA were used to compare groups, and *P*-values < 0.05 were considered statistically significant.

## Results

### The activation of CD11c^+^CXCR5^+^ DCs was comparable between WT and PD-1-deficient ITV-immunized mice

**Fig. 1:**
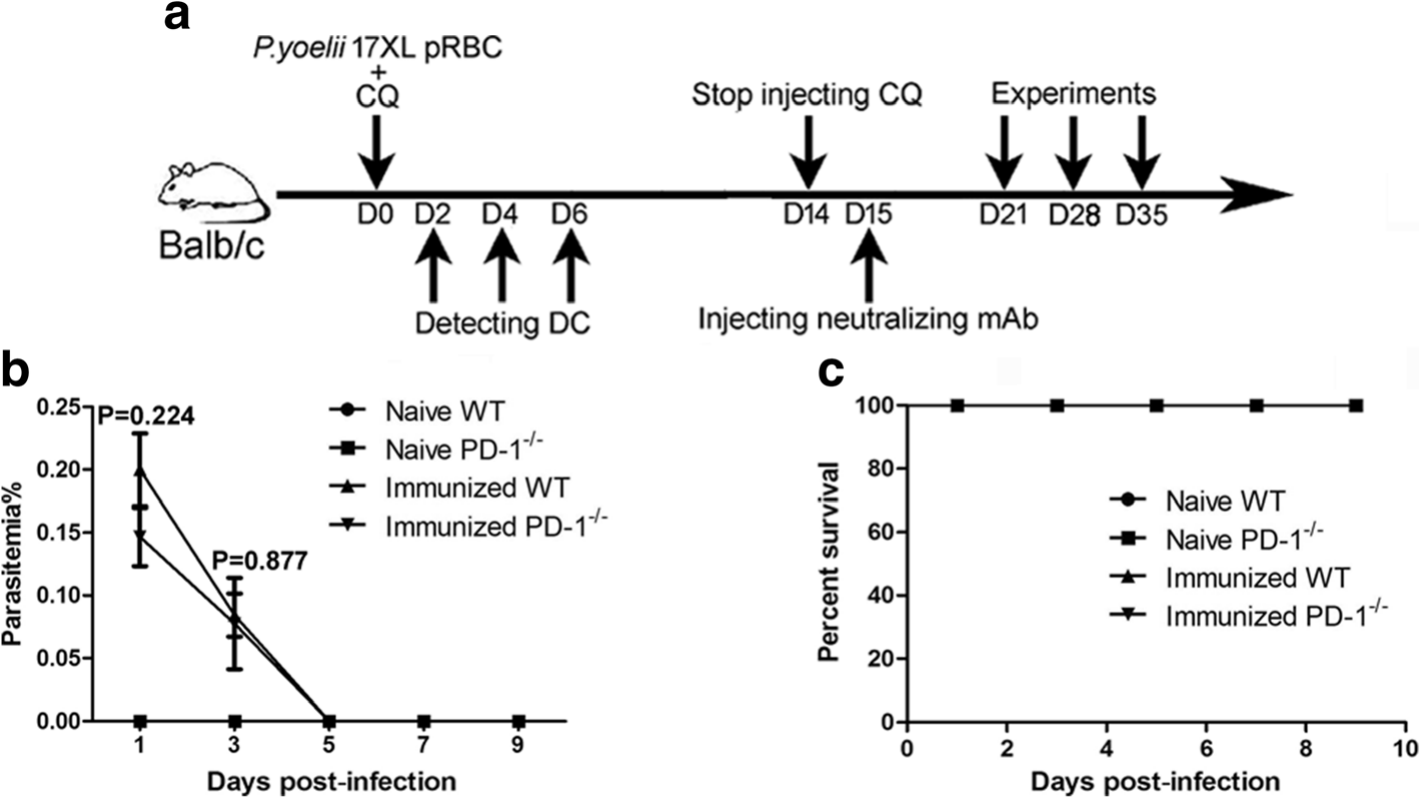
The parasitemia and survival rate in all treated mice from the beginning of CQ treatment. **a** The procedure for the ITV immunization and experiment operation. **b**, **c** WT (*n* = 5) or PD-1^-/-^ mice (*n* = 5) were intravenously (i.v.) injected with 10^6^ *P. yoelii* 17XL-infected red blood cells (RBCs) (*Py*-iRBCs) or a matching number of normal RBCs (nRBCs) (negative control). The parasitemia and survival rate were recorded from the beginning of CQ treatment. Three independent experiments were performed. The data are presented as the mean ± SD. Data were compared with the nonparametric Mann-Whitney test. *Abbreviation*: *n*, number of samples

**Fig. 2:**
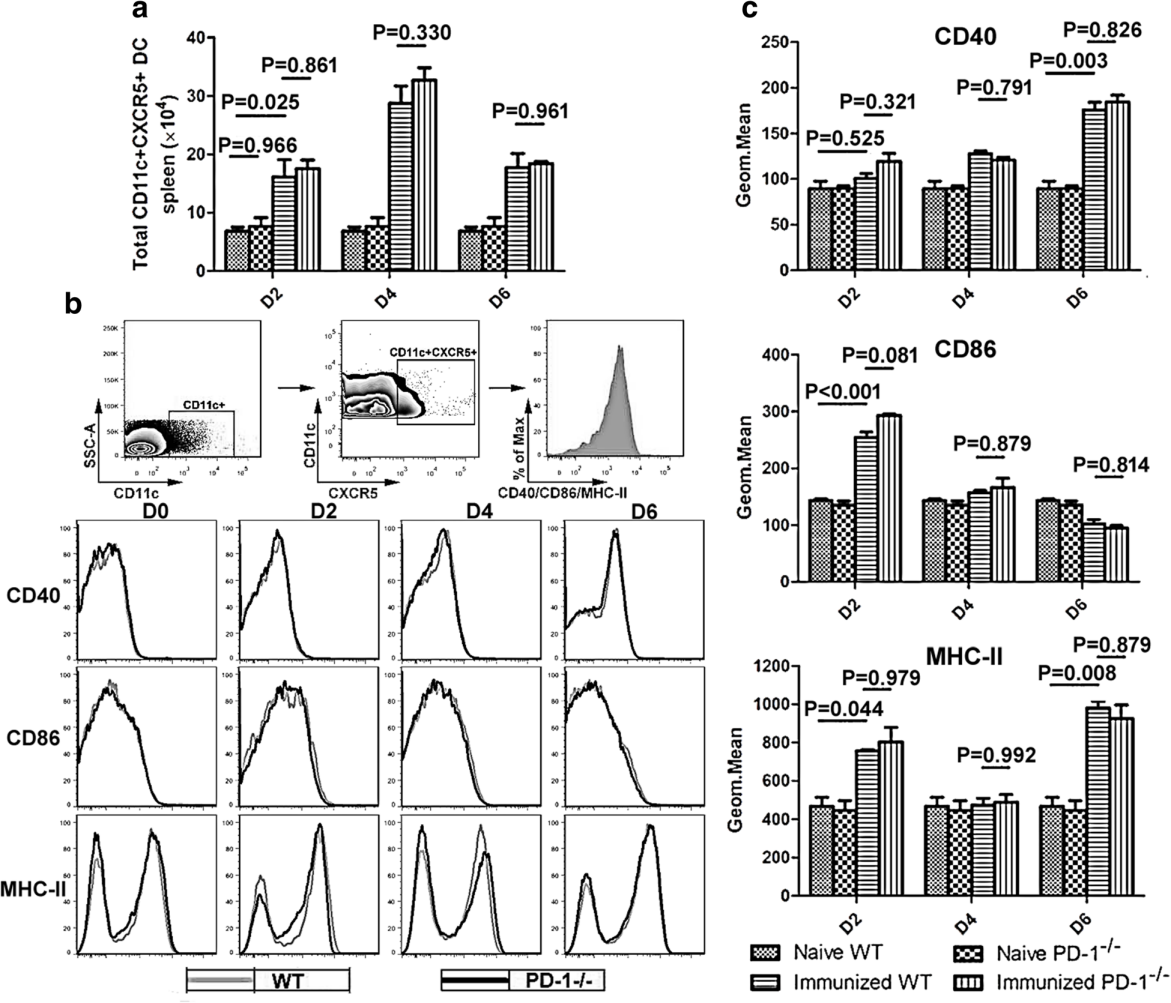
The total number and maturation phenotype of CD11c^+^CXCR5^+^ DCs were comparable between immunized WT and PD-1^-/-^ mice. **a-c** Splenocytes were isolated from the immunized WT (*n* = 5) and PD-1^-/-^ mice (*n* = 5) at 2, 4 and 6 days after the initial immunization. **a** Statistical analysis of the total number of CD11c^+^CXCR5^+^ DCs in the spleen from ITV-immunized WT and PD-1^-/-^ mice on days 2, 4 and 6 after the initial immunization. **b** Gating strategy and FACS analysis of the expression of CD40, CD86, and MHC-II on CD11c^+^CXCR5^+^ DCs from immunized WT (thin line) and PD-1^-/-^ (bold line) mice. Spleen lymphocytes were gated as CD11c^+^CXCR5^+^, and the expression levels of CD40, CD86 and MHC-II were analyzed. **c** A graphical representation of the geometrical mean of the immunofluorescence intensity of the expression of all three markers. Three independent experiments were performed. The data are presented as the mean ± SD. Data were compared with two-way ANOVA. *Abbreviation*: *n*, number of samples

First, the parasitemia (Fig. 1b) and survival rate (Fig. 1c) were recorded in all treated mice from the beginning of CQ treatment. As shown in Fig. 1, the parasitemia in all immunized mice appeared in day 1 and disappeared in day 5 post-infection and all mice survived during the process of CQ injection. Previous studies have found that CD11c^+^CXCR5^+^ DCs preferentially initiate TFH cell differentiation [13]. Therefore, the total number and maturation phenotype of CD11c^+^CXCR5^+^ DCs in the spleen were analyzed in ITV-immunized WT mice and PD-1^-/-^ mice 2, 4 and 6 days after the initial immunization. As shown in Fig. 2, the total number of CD11c^+^CXCR5^+^ DCs in the spleen was comparable between WT and PD-1^-/-^ mice, indicating no intrinsic defect in PD-1^-/-^ mice. Although immunized mice have greater number of CD11c^+^CXCR5^+^ DCs than naïve mice in the spleen (ANOVA: *F*_(3, 54)_ = 89.588, *P* = 0.025), there was no significant difference between the ITV-immunized WT mice and PD-1^-/-^ mice (Fig. 2a). At resting state, the expression of CD40, CD86, and MHC-II was similar between the WT and PD-1^-/-^ mice (Fig. 2b, c), indicating that there was no intrinsic DC defect in the absence of PD-1. Although the activation of CD11c^+^CXCR5^+^ DCs from immunized mice was significantly increased compared with DCs from naïve mice (ANOVA: *F*_(3, 54)_ = 33.831, *P* < 0.05), no significant differences in the expression of CD40, CD86, and MHC-II on the surface of CD11c^+^CXCR5^+^ DCs were detected between WT and PD-1^-/-^ immunized mice (Fig. 2b, c). Thus, these results suggest that the expansion of *Plasmodium*-specific TFH cells in ITV-immunized PD-1^-/-^ mice may not be attributable to enhanced DC function.

### The absence of PD-1 strongly increased the number of TFR cells in ITV-immunized mice

**Fig. 3:**
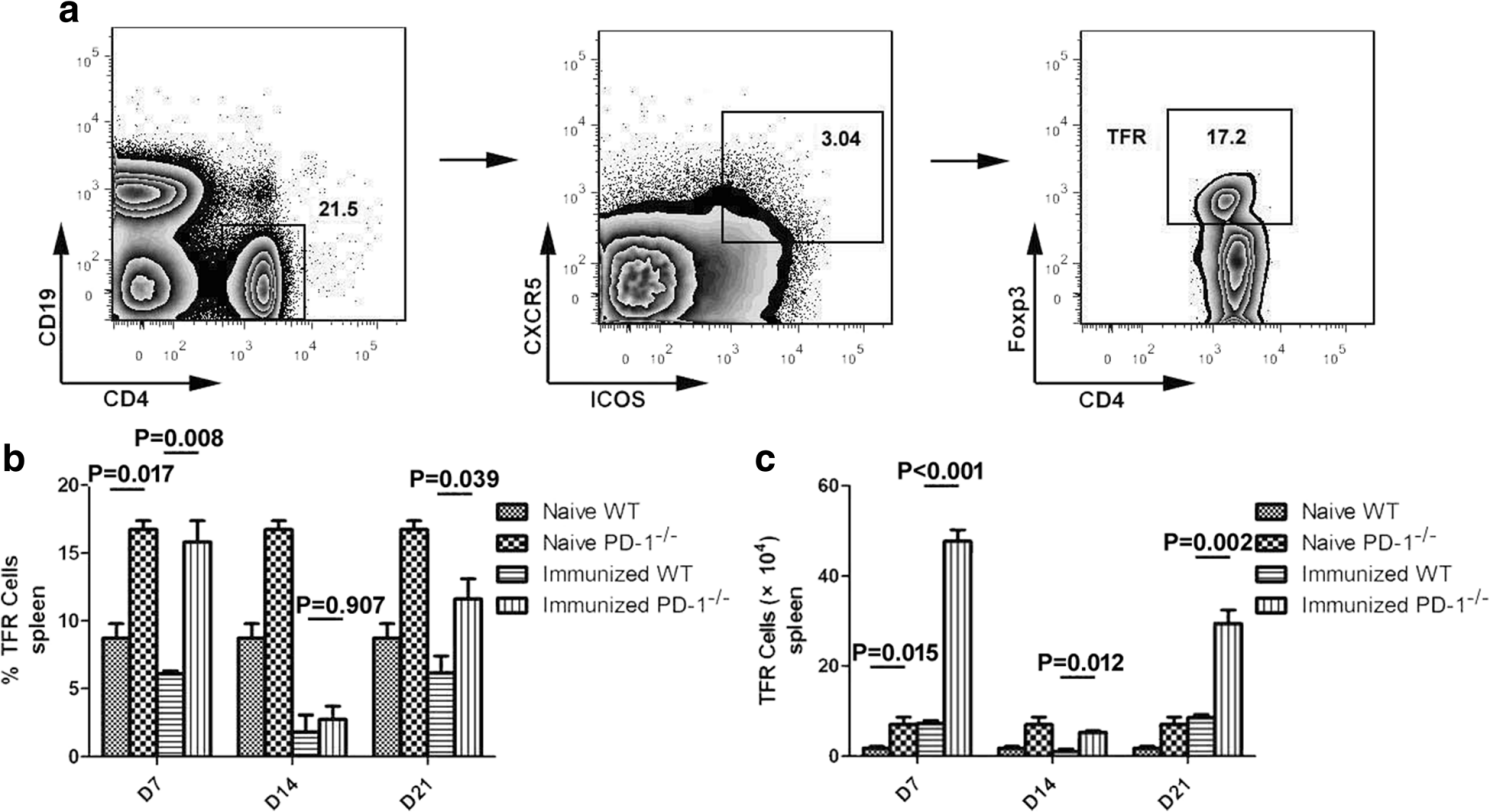
PD-1 deficiency contributed to the expansion of TFR cells in both naïve and immunized mice. Splenocytes were isolated from immunized WT (*n* = 5) and PD-1^-/-^ mice (*n* = 5) at the indicated times after the final immunization, and both the frequency and number of TFR cells were analyzed by FACS. **a** Representative FACS analysis of CD4^+^ICOS^+^CXCR5^+^Foxp3^+^CD19^−^ cells. **b**, **c** Statistical analysis of the frequency and number of TFR cells from ITV-immunized WT and PD-1^-/-^ mice on days 7, 14 and 21 after the final immunization. Three individual experiments were performed. The data are presented as the mean ± SD. Data were compared with the two-way ANOVA. *Abbreviation*: *n*, number of samples

TFR cells can inhibit humoral immunity by modulating TFH cell activity [17]. To test whether the increased expansion of TFH cells in ITV-immunized PD-1-deficient mice was a result of the inhibition of TFR cell activity, the frequency and number of splenic TFR cells were compared between immunized WT and PD-1^-/-^ mice. We defined TFR cells as CD4^+^ICOS^+^CXCR5^+^Foxp3^+^CD19^−^ as previously reported [18] and analyzed TFR cells on days 7, 14 and 21 after the final injection of CQ. The frequency and number of TFR cells in both ITV-immunized WT mice and PD-1^-/-^ mice gradually decreased from day 7 to 14 and then increased from day 14 to 21 after the final injection of CQ (Fig. 3b, c), suggesting that the immune-protection effect induced by ITV may be attributable to impaired TFR cell function. However, the frequency and number of TFR cells were much higher in PD-1^-/-^ naïve and immunized mice than in WT naïve and immunized mice after the final injection of CQ (ANOVA: *F*_(3, 54)_ = 55.609, *P* < 0.05) (Fig. 3b, c), which is consistent with a previous report [18]. Therefore, these data suggest that the expansion of *Plasmodium*-specific TFH cells is not due to a reduction in the number or frequency of TFR cells in ITV-immunized PD-1^-/-^ mice.

### The levels of IL-10, IFN-γ and MCP-1 were significantly increased in PD-1-deficient ITV-immunized mice

**Fig. 4:**
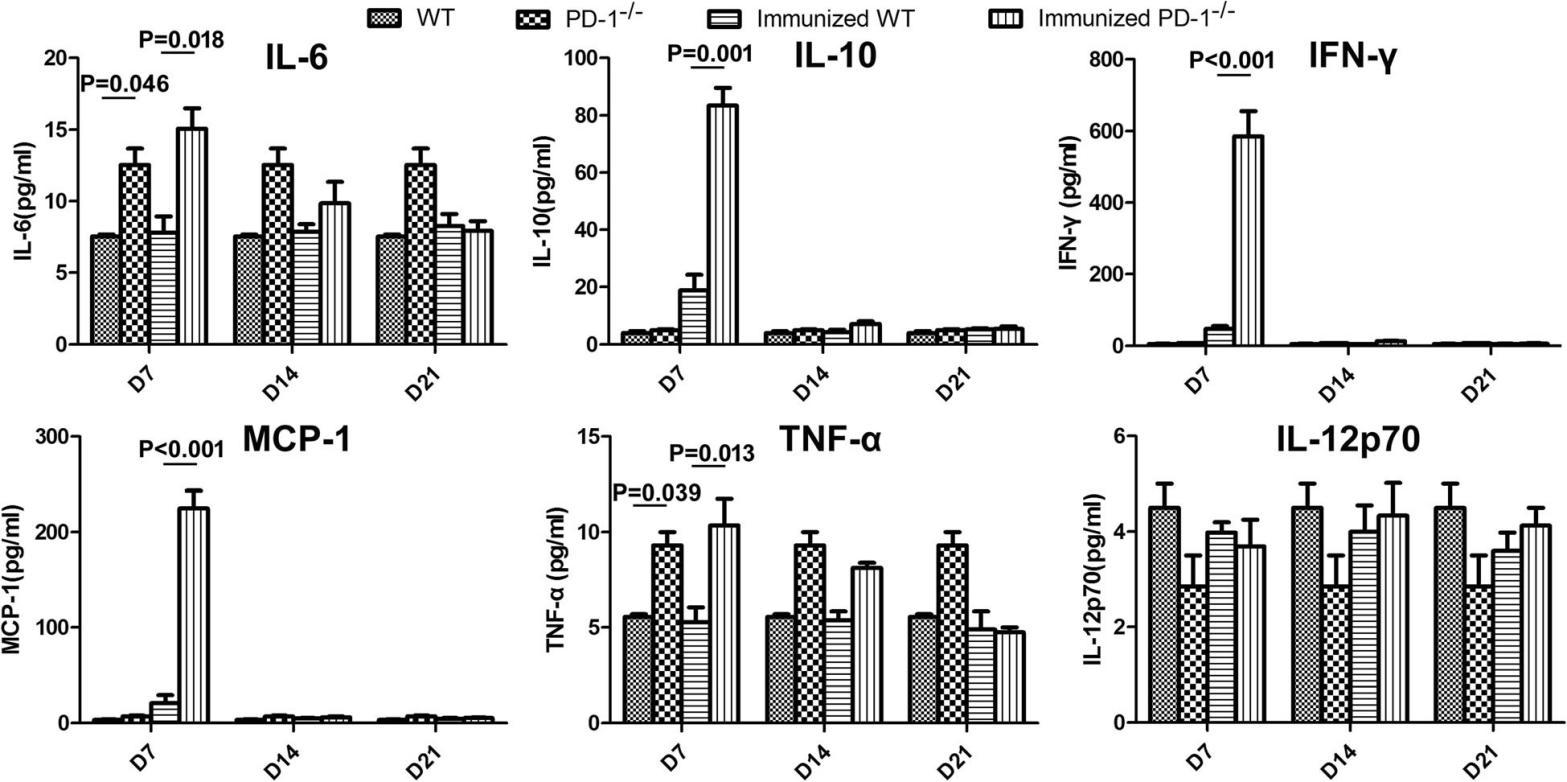
MCP-1, IFN-γ and IL-10 were significantly elevated in the serum of ITV-immunized PD-1^-/-^ mice. Serum samples were collected from immunized WT (*n* = 5) and PD-1^-/-^mice (*n* = 5) at the indicated times after the final immunization and the concentrations of cytokines were measured serially by a CBA. Three individual experiments were performed. The data are presented as the mean ± SD. Data were compared with the two-way ANOVA. *Abbreviation*: *n*, number of samples

Considering the important role of cytokines in supporting TFH cell differentiation [22, 23], we compared the serum cytokine level between the immunized WT and PD-1^-/-^ mice at 7, 14 and 21 days after the last CQ injection. The concentrations of IL-6, MCP-1, IFN-γ, TNF-α, IL-12p70 and IL-10 in serum were measured using a CBA. As shown in Fig. 4, the level of IL-12p70 was comparable between the two kinds of immunized mice. Although higher levels of IL-6 and TNF-α were detected in PD-1^-/-^ immunized mice than in immunized WT mice on day 7 after the final CQ injection, these cytokines were also higher in PD-1^-/-^ naïve mice than in WT naïve mice. Interestingly, although no significant difference was found at either day 14 or 21 after the final injection of CQ, significant increases in MCP-1 (224.89 ± 18.56 *vs* 20.91 ± 8.56 pg/ml; ANOVA: *F*_(3, 54)_ = 9.059, *P* < 0.001), IFN-γ (585.64 ± 70.38 *vs* 48.58 ± 6.82 pg/ml; ANOVA: *F*_(3, 54)_ = 9.158, *P* < 0.001) and IL-10 (83.45 ± 6.06 *vs* 18.75 ± 5.5 pg/ml; ANOVA: *F*_(3, 54)_ = 10.258, *P* = 0.001) in PD-1^-/-^ immunized mice were observed on day 7 after the final injection of CQ (Fig. 4), indicating that these cytokines may be involved in *Plasmodium*-specific TFH cell differentiation. Thus, these data suggest that increased levels of MCP-1, IFN-γ and IL-10 may promote *Plasmodium*-specific TFH cells expansion.

### Elevated cytokine levels substantially contributed to the expansion of TFH cells and GC B cells in immunized PD-1^-/-^ mice

**Fig. 5:**
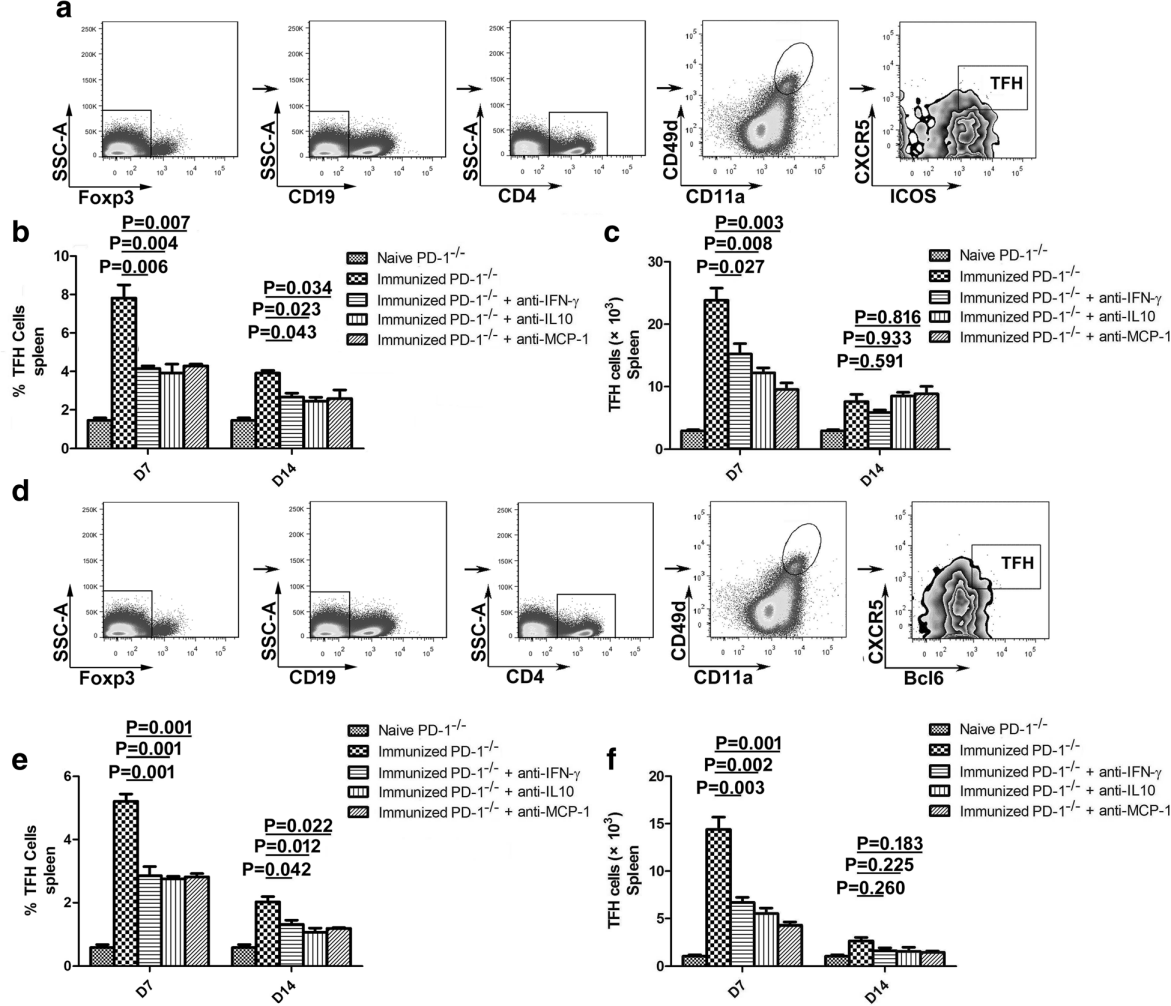
Frequency and number of *Plasmodium*-specific TFH cells in the spleens of ITV-immunized WT and PD-1^-/-^ mice. Splenocytes were isolated from immunized WT (*n* = 5) and PD-1^-/-^ mice (*n* = 5) on days 7 and 14 after the final immunization and the *Plasmodium*-specific TFH cells were analyzed by FACS. **a** Representative FACS analysis of Foxp3^-^CD19^-^CD4^+^CD11a^+^CD49d^+^CXCR5^+^ICOS^+^ *Plasmodium*-specific TFH cells. **b**, **c** Statistical analysis of the frequency and number of *Plasmodium*-specific TFH cells (Foxp3^-^CD19^-^CD4^+^CD11a^+^CD49d^+^ CXCR5^+^ICOS^+^) from immunized WT and PD-1^-/-^ mice. **d** Representative FACS analysis of Foxp3^-^CD19^-^CD4^+^CD11a^+^CD49d^+^CXCR5^+^Bcl6^+^ *Plasmodium*-specific TFH cells. **e**, **f** Statistical analysis of the frequency and number of *Plasmodium*-specific TFH cells (Foxp3^-^CD19^-^CD4^+^CD11a^+^CD49d^+^CXCR5^+^Bcl6^+^) from immunized WT and PD-1^-/-^ mice. Three individual experiments were performed. The data are presented as the mean ± SD. Data were compared with two-way ANOVA. *Abbreviation*: *n*, number of samples

To determine whether elevated levels of cytokines (MCP-1, IFN-γ and IL-10) could promote TFH cells expansion in PD-1^-/-^ immunized mice, we performed blocking experiments using neutralizing mAbs against MCP-1, IFN-γ, or IL-10 beginning one day after the last CQ injection. The frequency and number of splenic *Plasmodium*-specific TFH cells were then compared between the two groups of immunized mice. According to a previous study, Foxp3^-^CD19^-^CD4^+^CD11a^+^ CD49d^+^CXCR5^+^ICOS^+^ or Foxp3^-^CD19^-^CD4^+^CD11a^+^ CD49d^+^CXCR5^+^Bcl6^+^ cells are considered to be *Plasmodium*-specific TFH cells [12, 18]. The *Plasmodium*-specific TFH cell frequency and number peaked on day 7 after the final CQ injection and then gradually decreased over time. Interestingly, compared with normal PD-1^-/-^ immunized mice, the frequency and number of Foxp3^-^CD19^-^CD4^+^CD11a^+^CD49d^+^ CXCR5^+^ICOS^+^ or Foxp3^-^CD19^-^CD4^+^CD11a^+^CD49d^+^CXCR5^+^Bcl6^+^ TFH cells decreased by nearly half in mice treated with blocking mAbs on day 7 after the final CQ injection (ANOVA: *F*_(4, 44)_ = 19.208, *P* < 0.05), although no significant difference in the number of *Plasmodium*-specific TFH cells was found on day 14 after the final CQ injection (Fig. 5b, c, e, f).

**Fig. 6:**
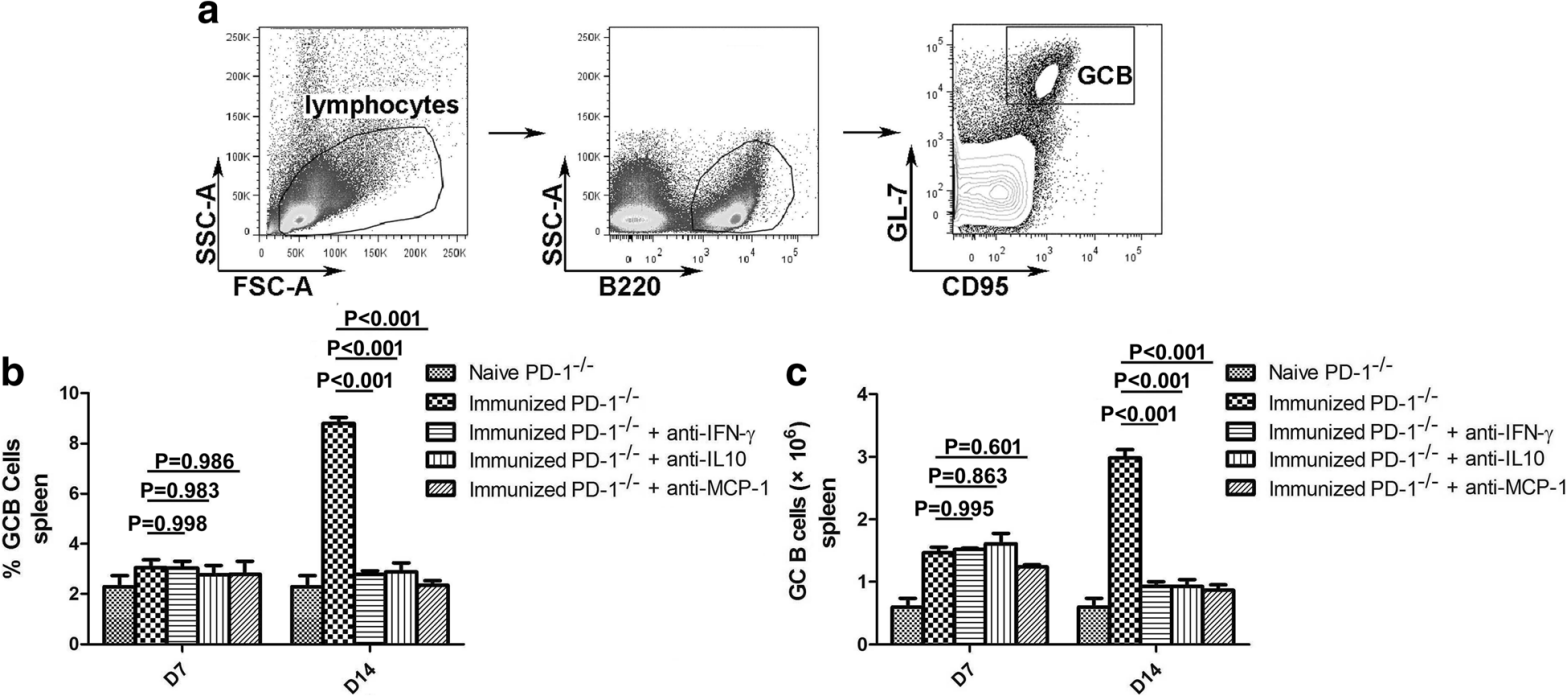
Frequency and number of GC B cells in the spleens of ITV-immunized WT and PD-1^-/-^ mice. Splenocytes were isolated from immunized WT (*n* = 5) and PD-1^-/-^ mice (*n* = 5) on days 7 and 14 after the final immunization and both the frequency and number of GC B cells were analyzed by FACS. **a** Representative FACS analysis of B220^+^GL-7^+^CD95^+^ GC B cells. **b**, **c** Statistical analysis of the frequency and number of GC B cells from ITV-immunized WT and PD-1^-/-^ mice. Three individual experiments were performed. The data are presented as means ± SD. Data were compared with two-way ANOVA. *Abbreviation*: *n*, number of samples

The most important biological function of TFH cells is to aid GC B cells in the generation of GCs and long-term protective humoral responses [1]. Thus, to further confirm the roles of MCP-1, IFN-γ and IL-10 in the regulation of TFH cell expansion, the frequency and number of GC B cells in the spleen were also detected on days 7 and 14 after the final CQ injection. As shown in Fig. 6, the GC B cells frequency and number decreased significantly in ITV-immunized PD-1^-/-^ mice after treatment with blocking mAbs on day 14 (ANOVA: *F*_(4, 44)_ = 12.790, *P* < 0.001), but no significant difference was found on day 7 after the final injection of CQ. Taken together, these results demonstrate that the cytokines MCP-1, IFN-γ and IL-10 substantially contribute to the expansion of TFH cells and GC B cells in immunized PD-1^-/-^ mice.

## Discussion

PD-1 and its ligands (PD-L1 and PD-L2) deliver inhibitory signals, which can regulate the balance between T cell activation and tolerance. Evidence has already confirmed that PD-1 is highly expressed on GC TFH cells and expected to provide an inhibitory signal to GC TFH cells, preventing excess CD4^+^ T cell proliferation in GC [16, 24]. However, the precise mechanisms by which PD-1 signaling modulates TFH cell activation remain unclear. Our previous study found that PD-1 deficiency substantially promoted the expansion of *Plasmodium*-specific TFH cells in ITV-immunized mice, which focused on the whole blood-stage malaria parasites [12]. However, the mechanism of *Plasmodium*-specific TFH cell activation in PD-1^-/-^ immunized mice remains to be elucidated. In this study, we demonstrated that the cytokines MCP-1, IFN-γ and IL-10 participate in the induction of *Plasmodium*-specific TFH cell expansion in PD-1^-/-^ immunized mice. Although recent studies have revealed that IFN-γ and IL-10 signaling can modulate TFH cell differentiation, reports regarding the function of IL-10 signaling in modulating TFH cell activation are contradictory [25–28]. Here, we found that IL-10 neutralization significantly inhibited *Plasmodium*-specific TFH cell proliferation in PD-1^-/-^ immunized mice. This is consistent with a previous study that also focused on pathogen-specific TFH cells instead of total TFH cells [25]. Thus, we hypothesized that IL-10 signaling could have differential effects on the differentiation of total TFH cells and that of pathogen-specific TFH cells. Studies have found that IFN-γ can act directly on TFH cells to promote their expansion [28], while IL-10 can act indirectly on T cells to substantially dictate the differentiation state and function of TFH cells [25]. Surprisingly, our results also showed that MCP-1 participates in *Plasmodium*-specific TFH cell expansion. To the best of our knowledge, this is the first study to reveal the relationship between MCP-1 and TFH cell differentiation. Evidence has confirmed that MCP-1 is mainly produced by monocytes/macrophages [29]. Thus, in addition to eliminating *Plasmodium*-infected RBCs (iRBCs) [30], monocytes/macrophages may also be involved in the regulation of humoral immunity *via* the secretion of MCP-1.

Another important issue is the cell types that secreted the MCP-1, IFN-γ and IL-10 observed in PD-1^-/-^ immunized mice. Although CD4^+^ T cells, CD8^+^ T cells, macrophages and DCs have been analyzed for their ability to secrete these cytokines (data not shown), we have not yet identified the major cell type producing MCP-1, IFN-γ and IL-10. It is possible that many types of cells secrete these cytokines in PD-1^-/-^ immunized mice. A recent study showed that PD-1 signaling deficiency can enhance the secretion of IL-10 and IFN-γ in allogenic cultures of CD4^+^ T cells with various DC populations [31], which may provide clues for further experiments. Meanwhile, our results showed that the elevated levels of MCP-1, IFN-γ and IL-10 in serum were observed on day 7 after the final injection of CQ. Considering the parasitemia in immunized mice appeared on day 6 and disappeared on day 12 after the final injection of CQ (Additional file 1: Figure S1), we hypothesized that this phenomenon may correlate with the complete elimination of iRBCs in ITV-immunized mice in the early stage of CQ withdrawal.

Recent evidence has suggested that the engagement of CXCR5 on DCs may preferentially condition them to directly prime TFH cell responses [1, 13]. However, unlike the maturation profiles and phenotype of DCs in mice immunized with the whole-killed *P. yoelii* 17XL vaccine (WKV) [20], we found that CXCR5^+^ DCs exhibited unique expression patterns of CD40, CD86 and MHC-II in ITV-immunized mice. The levels of CD40, CD86 and MHC-II on the CXCR5^+^ DCs surface were significantly higher in immunized mice than in naïve mice after the primary immunization with WKV. However, the expression level of CD40 gradually increased over time, and a significant difference between immunized mice and naïve mice only appeared on day 6 after the initial ITV immunization. In contrast to CD40, the expression of CD86 began to gradually decrease two days after the first immunization. In addition, differences in the expression of MHC-II were mainly apparent on day 2 and day 6 after the initial immunization. This is partially consistent with a recent study, which also found that each activated DC phenotype exhibited a different expression pattern during *P. yoelii* infection [32]. Thus, we hypothesized that pRBC lysates and live iRBCs may affect the phenotypic and functional maturation of DCs *via* different mechanisms. To the best of our knowledge, up to now there has not been any report concerning the relationship between the PD-1 signal and CXCR5^+^ DC activation. Our results showed that the total number of CD11c^+^CXCR5^+^ DCs in the spleen was comparable between WT and PD-1^-/-^ mice, immunized WT and immunized PD-1^-/-^ mice, which suggests that PD-1 signal may not be involved in the regulation of CXCR5^+^ DC activation.

TFR cells are a subset of Foxp3^+^ Tregs, which were found within GC and suppress the magnitude and longevity of TFH cells and GC responses [1, 33]. TFR cells express many canonical TFH cell molecules, including PD-1, CXCR5, ICOS and Bcl6 and Foxp3 is currently considered to be the most important marker that distinguishes TFR cells from TFH cells [18]. Therefore, Foxp3^-^ cells were selected for further analyses of TFH cells in our study. Meanwhile, studies have found that the integrins CD49d and CD11a on antigen-experienced CD4^+^ T cells could be used to identify *Plasmodium*-specific CD4^+^ T cells [19], and therefore Foxp3^-^CD19^-^CD4^+^CD11a^+^CD49d^+^CXCR5^+^ICOS^+^ or Foxp3^-^CD19^-^CD4^+^CD11a^+^ CD49d^+^CXCR5^+^Bcl6^+^ cells were regarded as *Plasmodium*-specific TFH cells in our study. Although our results also confirmed that PD-1 signaling decreased the number of TFR cells, the precise regulatory mechanism of TFR cell activation remains largely unknown. A recent study has found that PD-L1, one of the PD-1 ligands which are expressed on DC, is essential for limiting TFR cell differentiation [34]. These results may provide new ideas for further research. In addition, we found that the frequency and number of TFR cells significantly decreased from day 7 to 14 and gradually increased from day 14 to 21 after the final injection of CQ in ITV-immunized mice. Therefore, these results indicate that humoral immunity induced by ITV may be associated with the impaired function of TFR cells, which would have implications in the design of effective malaria vaccines.

## Conclusions

In summary, we demonstrated that PD-1 deficiency significantly increased the levels of MCP-1, IFN-γ and IL-10 in the serum of ITV-immunized mice. We further confirmed that these elevated cytokines substantially contributed to the expansion of *Plasmodium*-specific TFH cells and GC B cells in PD-1^-/-^ immunized mice. Thus, our findings not only have implications for understanding the activation mechanism of *Plasmodium*-specific TFH cells but also contribute to our knowledge regarding the rational design of an effective malaria vaccine.

## Additional file

Additional file 1: **Figure S1.** The parasitemia in ITV-immunized mice after the final injection of CQ. After the last CQ injection, the parasitemia was recorded in four groups. The data are presented as the mean ± SD. Data were compared with the nonparametric Mann-Whitney test. (TIF 4538 kb)

## Abbreviations

CQ: Chloroquine
DCs: Dendritic cells
GCs: Germinal centers
ICOS: Inducible T-cell co-stimulator
iRBCs: *Plasmodium*-infected RBCs
ITV: Infection treatment vaccine
MCP-1: Monocyte chemoattractant protein-1
MHC-II: MHC class II
PD-1: Programmed death 1
*Py*-iRBCs: *P. yoelii* 17XL-infected red blood cells
TFH cells: T follicular helper cells
TFR cells: Follicular regulatory T cells
WKV: Whole-killed *P. yoelii* 17XL vaccine
