# Supplementary figures and images for: PD-1 deficiency promotes TFH cells expansion in ITV-immunized mice by upregulating cytokines secretion
Source: Parasit Vectors. 2018 Jul 6;11:397. doi: 10.1186/s13071-018-2984-4 (PMC6035468; doi:10.1186/s13071-018-2984-4)

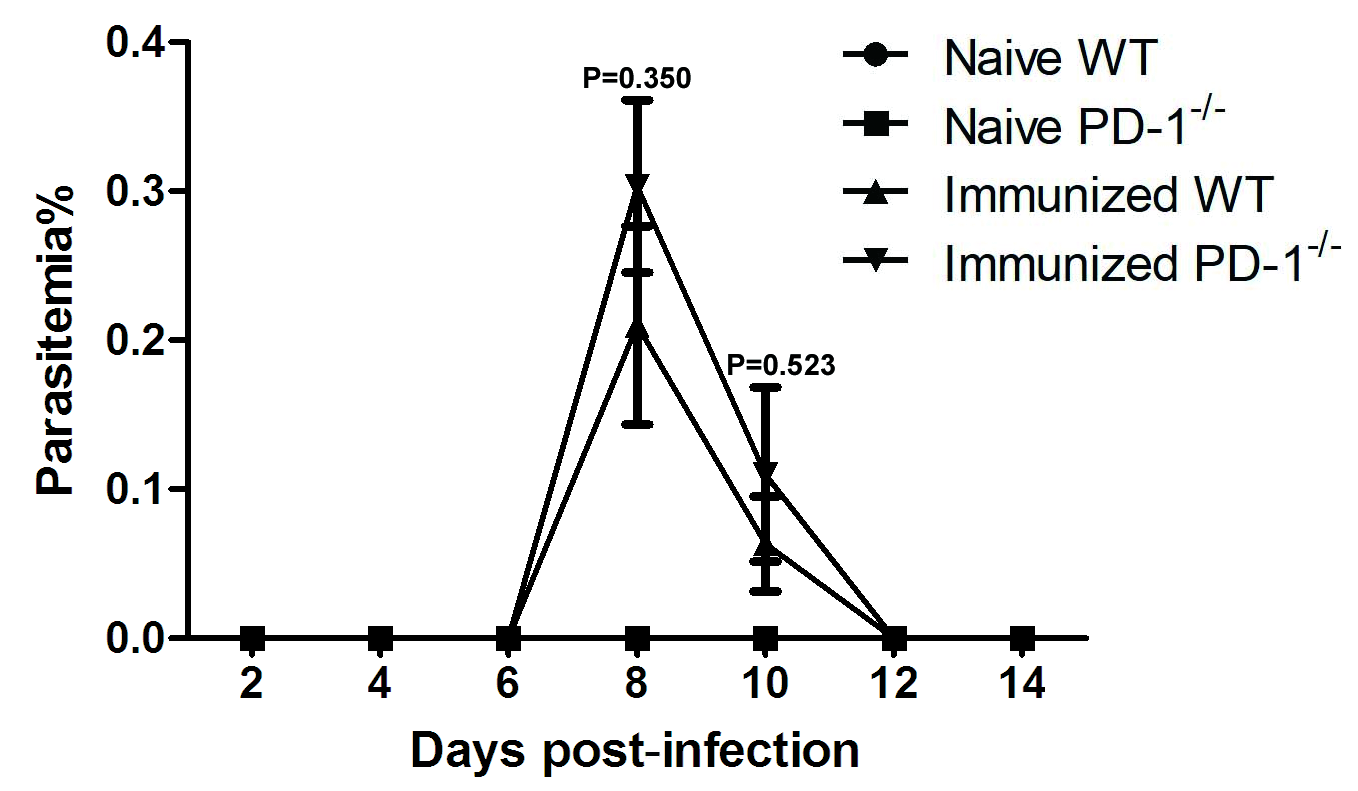

Supplement: Supplementary file 1 — Figure S1. The parasitemia in ITV-immunized mice after the final injection of CQ. After the last CQ injection, the parasitemia was recorded in four groups. The data are presented as the mean ± SD. Data were compared with the nonparametric Mann-Whitney test. (TIF 4538 kb) [file 13071_2018_2984_MOESM1_ESM.tif]
